# The “basics” of HIV-1 assembly

**DOI:** 10.1371/journal.ppat.1011937

**Published:** 2024-02-01

**Authors:** Christopher Sumner, Akira Ono

**Affiliations:** Department of Microbiology and Immunology, University of Michigan Medical School, Ann Arbor, Michigan, United States of America; University of Iowa, UNITED STATES

## Introduction

**Fig 1:**
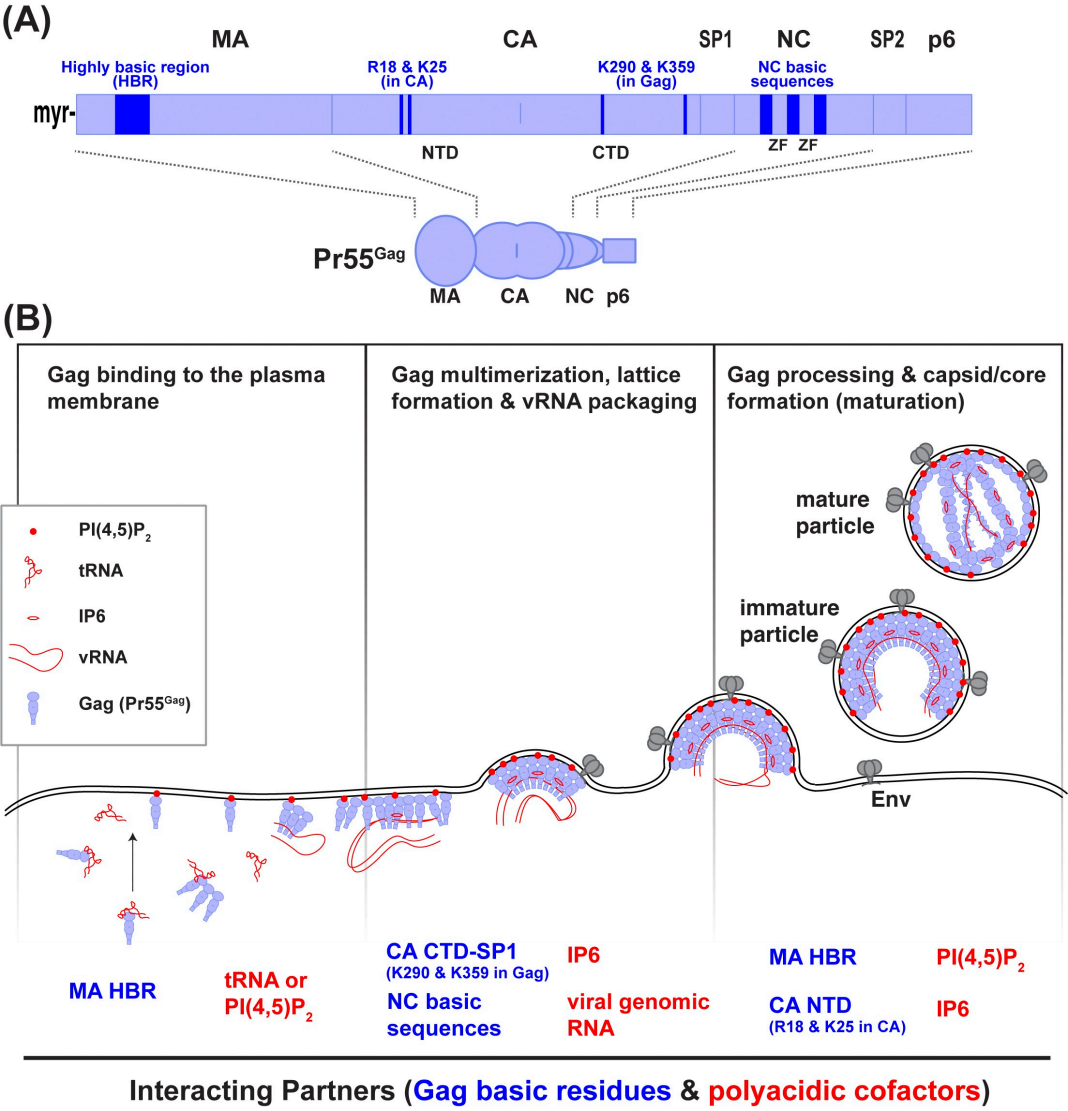
The schematic representations of HIV-1 Gag domains and assembly process. (A) Gag domains and key basic residues or basic residue clusters discussed in this review. Upon cleavage of Pr55^Gag^, individual domains give rise to mature Gag proteins, MA, CA, p2 (SP1), NC, p1 (SP2), and p6. NTD, N-terminal domain; CTD, C-terminal domain; ZF, zinc finger. (B) Interactions between basic residues in Gag and polyanions play important roles at different steps of virus particle formation and post-assembly processes. In some cases, the basic residues in Gag and the polyanions switch their interaction partners through the transitions from one step to another. PI(4,5)P_2_, phosphatidylinositol (4,5) bisphosphate; IP6, inositol hexakisphosphate; vRNA, viral genomic RNA.

Retrovirus particle assembly is driven by a structural protein, Gag (for a comprehensive review, see [1]). HIV-1 Gag, synthesized as the 55 kDa precursor polyprotein Pr55^Gag^, contains matrix (MA), capsid (CA), nucleocapsid (NC), and p6 domains as well as 2 spacer peptides (SP1 and SP2) (Fig 1A). Early in the viral assembly process, MA drives binding of Gag specifically to the plasma membrane where CA and NC promote the multimerization of Gag to form an immature lattice [1]. Growth of the immature lattice induces membrane curvature at the site of assembly, eventually leading to budding of the immature viral particle. Host ESCRT proteins, recruited by the p6 domain, promote the release of the nascent viral particle from the cell surface [1]. During progeny virus production, Gag undergoes multiple protein–protein interactions with each other and with host proteins like ESCRT proteins. However, Gag also interacts with various non-proteinaceous polyacidic molecules via basic amino acid clusters within MA, CA, and NC domains (Fig 1B). Below, we will focus on 5 key points regarding these Gag-polyanion interactions and discuss how they regulate HIV-1 assembly.

## MA highly basic region (HBR) interacts with acidic phospholipids, thereby mediating Gag-membrane binding

The HIV-1 MA domain contains 2 major membrane-binding signals, an N-terminal myristoyl moiety and a conserved basic amino acid cluster spanning the MA residues 15–31 known as the highly basic region (HBR). Early studies showed that the HBR promotes specific targeting of Gag to the plasma membrane enabling efficient release of viral particles [2,3] and that the MA N-terminal sequence containing the HBR confers upon heterologous proteins the ability to bind lipid membranes containing acidic phospholipid phosphatidylserine (PS) [4]. Early structure analyses of purified MA protein suggested that the MA-HBR serves as an interface with acidic phospholipids in the cytoplasmic leaflet of the plasma membrane [5].

The acidic phospholipid that determines specific localization of Gag in cells is phosphatidylinositol-(4,5)-bisphosphate [PI(4,5)P_2_]. This highly negatively charged lipid is present predominantly in the inner leaflet of the plasma membrane. Depletion of cellular PI(4,5)P_2_ prevents Gag from binding to the plasma membrane and drastically reduces HIV-1 particle production [6]. In vitro studies showed that Gag binds PI(4,5)P_2_-containing membranes more efficiently than membranes containing PS at the matching level of total negative charge or membranes containing PI (3,5)P_2_ in place of PI(4,5)P_2_, suggesting that the interaction between MA and PI(4,5)P_2_ is not solely electrostatic [7–10]. Indeed, the interaction of Gag with the head group of PI(4,5)P_2_ involves specific basic residues within the HBR [9–12]. Acute depletion of cellular PI(4,5)P_2_ leads to detachment of pre-budding Gag lattices, i.e., Gag that is already membrane-bound, from the plasma membrane [13], suggesting that the MA-PI(4,5)P_2_ interaction is reversible and must be maintained during the assembly process.

## MA-HBR also interacts with tRNA, which regulates membrane binding of Gag

In addition to acidic phospholipids, the MA-HBR binds RNA [14–19]. This binding prevents Gag membrane binding in general; however, full-length Gag can bind membranes containing PI(4,5)P_2_, which outcompetes RNA for MA binding [20]. The RNA bound to the MA domain in the cytosol of Gag-expressing cells is primarily tRNA [19]. In vitro studies showed that tRNA prevents Gag from binding membranes containing PS, which is ubiquitously present in various organelle membranes [21–23]. These observations support a model that in cells, RNA binding to the MA-HBR prevents Gag association with non-PI(4,5)P_2_-containing membranes, while allowing for Gag binding to the plasma membrane, which contains PI(4,5)P_2_. Thus, tRNA enhances the PI(4,5)P_2_ specificity of the MA-HBR. Additionally, based on the structure of a tRNA-MA complex, it is predicted that Gag lattice growth may require tRNA-MA dissociation although this remains to be experimentally tested [15].

PI(4,5)P_2_ and other acidic phospholipids promote assembly of other enveloped viruses beyond retroviruses, including filoviruses [24] and paramyxoviruses [25]. Although it remains to be examined whether membrane binding of filo- and paramyxovirus matrix proteins is regulated by competition between acidic phospholipids and RNA like HIV-1 MA, such competition has been observed for poliovirus 3CD protein. This protein contains a cluster of basic residues, which bind to RNA and the acidic phospholipids [PI4P and PI(4,5)P_2_] in a mutually exclusive manner [26].

## CA-IP6 interactions promote HIV-1 immature lattice formation

After plasma membrane binding HIV-1 Gag undergoes higher-order multimerization to form the immature lattice, which leads to formation of immature virus particles. The process of lattice formation is primarily driven by the CA domain, which promotes protein–protein interactions via dimer, trimer, and hexamer interfaces. The bundle consisting of the CA-CTD (C-terminal domain)-SP1 region of 6 Gag molecules is the basis for the hexameric pattern of the immature lattice. This bundle formation is facilitated by a polyanion, inositol hexakisphosphate (IP6), which is the most abundant cellular inositol phosphate derivative [27]. Early in vitro Gag assembly studies identified IP6 as a cofactor that allows Gag to form particles with the size similar to that of virus particles (approximately 100 nm) in the presence of nucleic acids, which would otherwise form particles with diameters of 25 to 30 nm [28]. Later studies revealed that IP6 enhances the in vitro assembly of truncated CA-SP1-NC or CA-SP1 proteins into immature lattices. This effect is due to binding of IP6 to a space located at the center of the six-helix bundle consisting of the CA-CTD-SP1 region, which is lined by 2 rings of lysine residues, K290 and K359 [29]. IP6 increases immature capsid assembly to a greater degree than other polyanions with similar geometry but different charge distribution [30]. Reducing the IP6 concentration in the cytoplasm, especially near the plasma membrane, diminishes HIV-1 assembly in cells [31–34]. Substituting both K290 and K359 with neutral amino acid residues alleviates the need for IP6 in immature particle formation, suggesting that IP6 prevents destabilization caused by electrorepulsion between CA-CTD-SP1 helices [32,35].

## The NC-RNA interaction promotes HIV-1 Gag multimerization

The NC domain of HIV-1 Gag recognizes the packaging signal in the viral genomic RNA via its zinc finger motifs, which promotes the selective viral genome packaging [36]. In addition, the polynucleotide binding ability of the NC domain mediated by basic residues surrounding the zinc fingers contributes to efficient assembly of the immature lattice independent of the zinc fingers or the viral RNA packaging signal [37–41]. In vitro assembly of purified Gag proteins into virus-like particles can be promoted even by oligodeoxyribonucleotides [28]. These observations support the role played by the NC basic residues in general RNA binding, which in turn promotes Gag multimerization. HIV-1 packages cellular mRNA species in the absence of viral RNA containing the packaging signal [42], suggesting that nonspecific RNA binding can replace viral genomic RNA for the role in virus assembly. Early in vitro assembly studies [43,44] as well as more recent studies using single particle tracking and computational modeling [45,46] suggest that RNA serves as a scaffolding for Gag due to its polyacidic nature, thereby limiting diffusion of Gag and promoting Gag multimerization. However, accumulating evidence also supports the role specifically for viral genomic RNA binding in nucleating Gag assembly [47,48].

Of note, the basic residues of NC can also bind membrane containing acidic phospholipids in vitro [49,50]. Such lipid interactions, which compete with the binding to RNA, are implicated in recruitment of ESCRT machinery [51].

## The Gag-polyanion interactions play additional roles after immature particle assembly

Upon release of assembled particles from infected cells, HIV-1 protease incorporated in the particles cleaves Pr55^Gag^, which triggers dissociation of CA and the downstream domains from the MA lattice and assembly of mature capsid consisting of the CA hexamers and pentamers (“maturation”). Some of the polyanions that facilitate immature particle assembly are present in these particles and perform additional roles after maturation.

HIV-1 particles are enriched in PI(4,5)P_2_ in the MA-dependent manner [52,53]. Recent cryoEM comparison of MA lattices in immature and mature particles revealed a structural change in MA-PI(4,5)P_2_ binding modes although whether this rearrangement of MA-PI(4,5)P_2_ interactions has a function in HIV-1 infection remains to be studied [54]. Most tRNAs bound to MA-HBR are thought to dissociate from MA upon membrane binding although we cannot rule out the possibility that some tRNAs are packaged into virions through direct binding to MA-HBR. However, incorporation of tRNA^Lys^, which serves as the primer for reverse transcription in the new replication cycle, is mediated by a mechanism mapped to CA [55]. In contrast to polyacidic ligands that bind MA-HBR, those that bind CA or NC have more clearly documented roles in post-maturation steps. During mature capsid formation, IP6 incorporated via interactions with K290 and K359 in Gag dissociates from the CA-CTD six-helix bundle and binds to the channels formed in CA-NTD hexamers and pentamers [29,56]. This relocated IP6 promotes stability of the mature viral capsid/core [31,57]. Finally, viral genomic RNAs incorporated via interactions with NC serve as the template for reverse transcription.

Binding to polyanions discussed above via basic amino acid clusters not only allows HIV-1 Gag to achieve a sufficiently high concentration for self-assembly but also ensures proper localization for exit and efficient genome packaging. Some polyanions play additional roles in the new infection cycle. The intricate process of interactions between the polyanions and Gag basic residue clusters, which includes switching of interaction partners, may offer many opportunities for interference (Fig 1). Therefore, the Gag interfaces for polyanions may serve as targets for antiviral therapeutics, like the protein–protein interface in Gag/CA targeted by the recently FDA-approved antiretroviral lenacapavir [58,59].
